# Early Versus Late Bowel Preparation for Suspected Acute Diverticular Bleeding: A Retrospective Study

**DOI:** 10.7759/cureus.86746

**Published:** 2025-06-25

**Authors:** Syed Rahman, Kyle Schneider, Hassan Allahrakha, Juan Carlos Barrera Gutierrez, Bryce Bushe, Prashant Kedia

**Affiliations:** 1 Internal Medicine, Methodist Health System, Dallas, USA; 2 Performance Improvement, Methodist Health System, Dallas, USA; 3 Methodist Digestive Institute, Methodist Health System, Dallas, USA; 4 Gastroenterology, Baylor Scott &amp; White Medical Center, Round Rock, Round Rock, USA; 5 Gastroenterology, Methodist Health System, Dallas, USA

**Keywords:** bowel prep, colonoscopy, diverticular bleed, gi bleed, resource utilization

## Abstract

Introduction

Diverticular bleeding (DB) is a significant cause of inpatient gastrointestinal (GI) bleeding. We hypothesized that early bowel preparation (EBP) administration would reduce hospital resource utilization and improve outcomes compared to late administration.

Methods

We retrospectively identified patients admitted with acute DB from January 2019 to July 2023. EBP was given within 24 hours of admission, and late bowel preparation (LBP) was given after 24 hours. Variables examined included length of stay, readmissions for GI bleed, packed red blood cell (pRBC) transfusions, days to resolution, colonoscopy, tagged red blood cell (RBC) scan, and computed tomography (CT) angiographies, using chi-squared and T-tests for statistical analysis.

Results

One hundred and thirty-four patients were included: EBP (n=51), LBP (n=26), and no bowel preparation (NBP) (n=57). There were no statistically significant differences in baseline variables between groups that received bowel preparation and those who did not. EBP patients underwent fewer colonoscopies (56.86% vs. 84.62%, P=0.01). They had shorter hospital stays (mean 4.82 vs. 5.27 days), faster time to resolution (mean 2.94 vs. 3.58 days), and fewer CT angiograms (19.61% vs. 23.08%), although not statistically significant. EBP patients underwent more tagged RBC scans (37.25% vs. 30.77%, P=0.5727). Readmission rates, number of pRBC transfusions, hemostasis during colonoscopy, and total imaging showed no difference.

Conclusion

EBP compared to LBP was associated with significantly fewer colonoscopies and a trend toward shorter hospital stays and resolution times, without impacting clinical outcomes such as readmission rates or transfusion needs. The reduction in colonoscopies in the EBP group suggests that EBP may allow clinicians to identify resolved DB quicker and facilitate discharge without unnecessary colonoscopy.

## Introduction

Diverticulosis is a common cause of lower gastrointestinal (GI) bleeding [1]. The estimated incidence of diverticular bleeding (DB) events in the United States represents a significant disease burden, with an incidence rate as high as 30.4 per 100,000 persons. The majority of DB are self-limited, and identification of the culprit lesion endoscopically is rare. DB hospitalizations may be prolonged due to the clinical challenge of distinguishing ongoing active hematochezia from old blood being passed through the colon.

The American College of Gastroenterology recommends colonoscopy as the initial diagnostic modality for nearly all patients presenting with acute lower GI bleeding [2]. However, with approximately 75% of DB events resolving spontaneously, the procedure often does not localize a specific source of bleeding at the time of the procedure or change the clinical course [3]. Additionally, there may be considerable provider variability in the management of acute DB.

In this study, we examine the possible cathartic benefits of early routine bowel preparation administration as compared to late administration in suspected acute DB cases as a measure to assess the rate of potential ongoing blood loss to make medical management decisions. We hypothesized that early decision-making may have profound implications for reduction in the use of healthcare resources for lower GI bleeding.

## Materials and methods

Study design

From January 2019 to July 2023, a retrospective study was conducted at a single center to assess the impact of early versus late administration of bowel preparation solution in patients with suspected acute DB. The study was conducted in compliance with the internal review board.

Inclusion and exclusion criteria

Inclusion criteria were as follows: age >18 years, evaluation by a GI consult service, and a diagnosis of "hematochezia," "acute gastrointestinal bleeding," “acute blood loss anemia,” or “anemia” with endorsed or witnessed episodes of painless hematochezia, which were suggestive of DB. The diagnosis of DB was made based on clinical presentation, with or without a history of known diverticular disease. Patients with GI malignancy, colonic mass or lesions, and a prolonged hospital stay for a reason other than GI bleeding were excluded from the study.

Patient groups

Patients included in this study were divided into three groups based on the timing of bowel preparation solution administration: early bowel preparation (EBP), late bowel preparation (LBP), and no bowel preparation (NBP). Patients in the EBP group were administered bowel preparation solution within 24 hours of hospital presentation, patients in the LBP were administered it after 24 hours of presentation, and patients in the NBP group were monitored only and did not receive it during the hospital stay. The bowel preparation solution used was 4 liters of Polyethylene Glycol (PEG) 3350. The selection of patients for being in the EBP, LBP, or NBP group was at the discretion of the attending physician, based on the overall clinical picture and physician experience/preference, with no predefined criteria for group assignment. Given the retrospective nature of our study, there were no defined clinical criteria at the time of patient selection for each group.

Variables of interest

The following variables were compared between each of the groups: age, sex, use of anticoagulation, duration of GI bleed, length of hospital stay, total number of transfused pRBC units, whether colonoscopy was performed during admission and if any therapeutic hemostasis techniques were used, number of readmissions for GI bleeding within one month and within three months, need for interventional radiology (IR) embolization, and number of imaging studies (CT angiography, nuclear tagged RBC scan, and angiography).

Data collection

Data were extracted from the electronic medical record (EMR) in a retrospective manner by three of the authors (SHR, KS, and HA) and double-checked by another author (PK).

Statistical analysis

Continuous variables were described using the mean and standard deviation for normally distributed data, or median, minimum, and maximum values (min-max) for non-normally distributed data. T-tests or ANOVA (for comparing three or more means) with Bonferroni correction were used to compare differences between means, while the non-parametric Kruskal-Wallis test was applied for non-normal distributions. Categorical variables were summarized as percentages and compared using the Pearson chi-square test. P-values less than 0.05 were considered statistically significant. The data analysis was conducted with the Statistical Analysis System (SAS) OnDemand for Academics 9.4 (SAS Inc., Cary, NC, USA).

## Results

Patient characteristics

A total of 134 patients were included in the study with a distribution of 83 females (61.2%) and 51 males (38.1%), with a median age of 75. Of these, 69 patients (51.5%) were receiving anticoagulation or antiplatelet agents, while 65 (48.5%) were not. Eighteen patients (14.9%) were receiving proton pump inhibitors (PPIs) and 103 (85.1%) were not. Ninety-nine (73.9%) patients had known diverticular disease identified on prior imaging or colonoscopy. Baseline characteristics were consistent across each group and are summarized in Table 1.

**Table 1:** Baseline characteristics of patient population. EBP: early bowel preparation; LBP: late bowel preparation; PPI: proton pump inhibitor; CHF: congestive heart failure; CKD: chronic kidney disease (any stage); COPD: chronic obstructive pulmonary disease; HTN: hypertension; CAD: coronary artery disease; DM: diabetes mellitus (type 2). Antiplatelet includes aspirin, clopidogrel, and ticagrelor. Lung disease includes asthma and COPD. Values are presented as N (%). The chi-square test was used to calculate *P*-values, with values less than 0.05 is considered significant.

Characteristic	EBP	LBP	No bowel preparation	Chi-square value	P-value
Sex, female	29 (21.64%)	17 (12.69%)	37 (27.61%)	0.9022	0.6369
Median age, years	76	77.5	73		0.5342
PPI usage					
Yes	38 (31.40%)	20 (16.53%)	45 (37.19%)	0.6805	0.7116
Antiplatelet usage					
Yes	26 (19.40%)	14 (10.45%)	25 (18.66%)	0.9145	0.6325
Comorbidities					
CHF	14 (28.00%)	4 (14.29%)	12 (20.69%)		
CKD	15 (30.00%)	7 (25.00%)	16 (27.69%)		
Lung disease	9 (18.00%)	4 (14.29%)	6 (10.34%)		
HTN	39 (78.00%)	22 (78.57%)	40 (68.97%)		
CAD	14 (28.00%)	6 (21.43%)	17 (29.31%)		
DM	22 (44.00%)	10 (35.71%)	15 (25.86%)		

Primary outcomes

**Figure 1:**
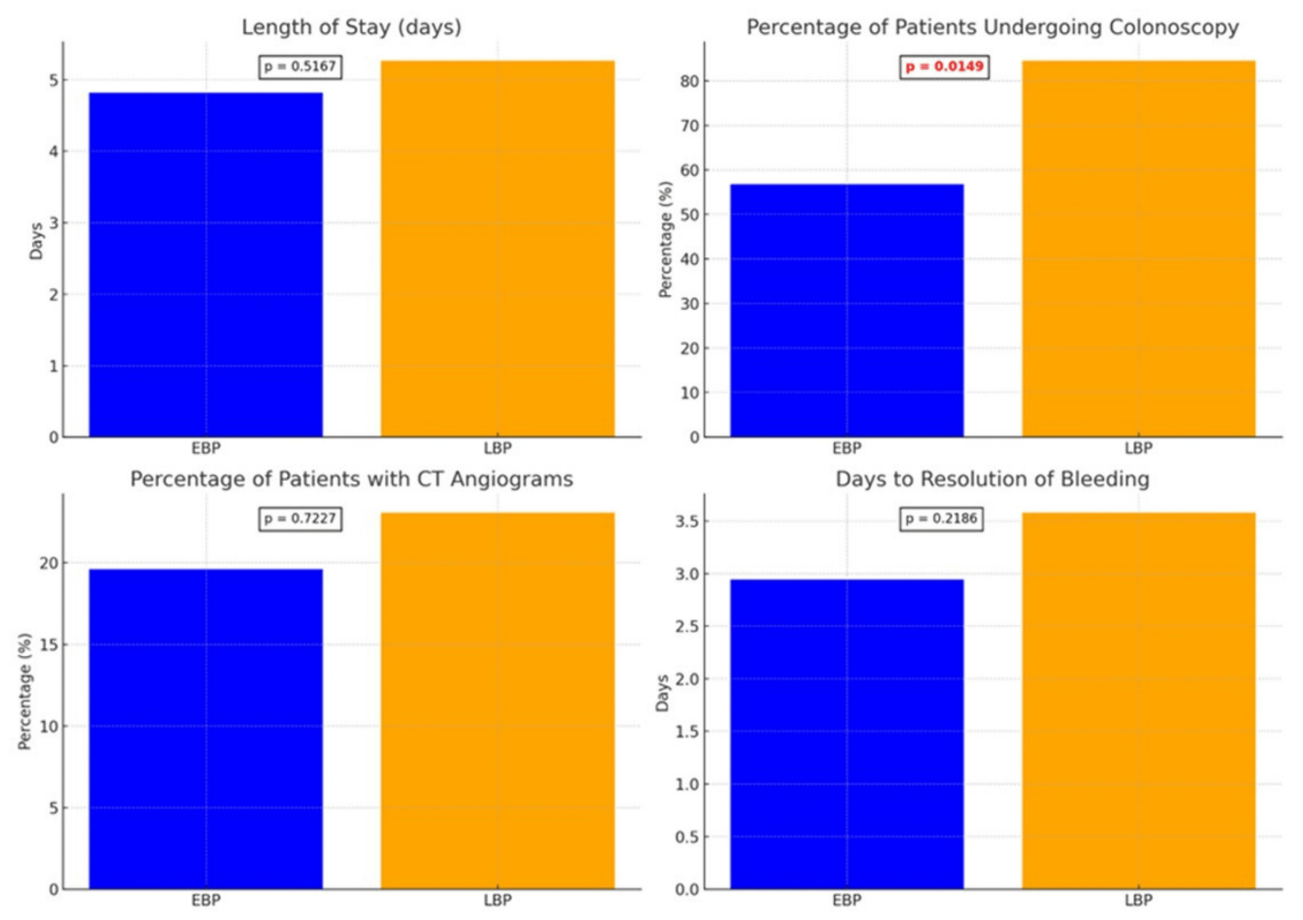
Comparison of hospital resource utilization. Variables compared include length of stay, number of inpatient colonoscopies, number of CT angiograms, and days to resolution of bleeding between the EBP and LBP groups. Values are listed in *x*-axis for each graph. The chi-square test was used to calculate P-values, with values less than 0.05 considered significant. CT: computed tomography; EBP: early bowel preparation; LBP: late bowel preparation.

EBP was initiated in 51 patients (38.1%), LBP in 26 patients (19.4%), and NBP in 57 patients (42.5%). Patients in the EBP group underwent fewer colonoscopies during admission compared to those in the late bowel preparation (LBP) group (56.9%, N=22 vs. 84.6%, N=22, P=0.0149). In comparison to the LBP group, patients in the EBP group also showed a trend towards shorter hospital stays (mean 4.82 vs. 5.27 days), faster time to resolution of bleeding (mean 2.94 vs. 3.58 days), and fewer computed tomography (CT) angiograms performed (19.61%, N=10 vs. 30.77%, N=6), although these were not statistically significant. Resolution of bleeding was defined as no more evidence of overt GI bleeding. We found that readmission rates, total number of pRBC transfusions required, and hemostasis during colonoscopy were similar between the groups. These results are summarized in Figure 1 and Table 2.

**Table 2:** Summary of results for EBP, LBP, and NBP groups. CT: computed tomography; pRBC: packed red blood cells; EBP: early bowel preparation; LBP: late bowel preparation; NBP: no bowel preparation. Values listed in *x*-axis for each graph. ANOVA and chi-square tests were used, with P-values less than 0.05 are considered significant. ^*^Patients in the NBP group did not undergo colonoscopy. ^**^Value listed is a chi-square value.

Variable	Early bowel preparation (n=51)	Late bowel preparation (n=26)	No bowel preparation (n=57)	ANOVA F ratio (chi-square value**)	P-value
Colonoscopies (%)	56.86	84.62	N/A*	5.9306**	0.0149
Length of stay (days)	4.82	5.27	4.25	1.53	0.2199
Time to resolution (days)	2.94	3.58	2.7	1.53	0.2216
CT angiogram (%)	19.61	23.08	21.05	1.5166	0.2232
Total pRBC transfused (mean)	2.08	2.88	2	1.04	0.3567
One-month readmit (%)	5.88	7.69	3.57	0.06	0.9363
Three-month readmit (%)	5.88	4	1.75	0.69	0.5056
Number of imaging studies performed (mean)	0.8	0.88	0.75	0.9	0.4085
Hemostasis during colonoscopy (%)	3.57	4.76	N/A*	0.0434**	0.8349

A total of 9 (18%) IR embolizations occurred in patients with EBP, compared to 4 (14%) with LBP and 7 (12%) with NBP. There were no statistically significant differences in any of the variables between the groups that received bowel preparation solution and those that did not. Patients who did not undergo bowel preparation had the same discharge criteria as those who did, namely resolution of clinical bleeding and stability of hemodynamic and hemoglobin levels.

## Discussion

Our results demonstrate that EBP administration upon presentation to the hospital in patients with suspected acute DB is associated with significantly fewer colonoscopies performed while inpatient compared to those that receive LBP administration. Although not statistically significant, there was a trend toward less utilization of hospital resources without any detriment to clinical patient outcomes in the EBP group compared to LBP group, suggesting potential benefit. Recent literature also suggests comparable outcomes with early colonoscopy (<24 hours) and delayed colonoscopy (>24 hours) for all inpatient lower GI bleeding admissions [4]. Current ACG guidelines recommend non-emergent inpatient colonoscopies, as urgent colonoscopies within 24 hours have not been shown to improve clinical outcomes. The guidelines also state that colonoscopy may not be needed in patients where bleeding has subsided and has had high-quality colonoscopy within the last 12 months. Our results support the suggestions of the guidelines that early colonoscopy within 24 hours may not provide clinical benefit, as we have demonstrated that EBP may result in the demonstration of resolution of DB within 24 hours and allow patients to follow up outpatient for colonoscopic evaluation [2]. Therefore, the concept of EBP in DB may provide an opportunity to decrease resource utilization and procedures (particularly the performance of colonoscopy) in these patients by allowing providers to assess the rate of ongoing bleeding earlier in the admission [5].

One of the most challenging aspects of managing patients with acute DB is determining whether bleeding has stopped or not [6]. Bowel cleansing can flush all the old blood from the colon without compromising hemodynamics or hemoglobin monitoring to provide an objective evaluation regarding ongoing bleeding. None of the patients in this study reported significant intolerance (severe nausea, vomiting, or inability to complete bowel preparation) to the bowel preparation. Generally, after bowel cleansing, patients who have stopped bleeding will have clear effluent from the colon as opposed to bright red hematochezia in patients who have ongoing bleeding. In contrast, not administering bowel preparation solution early in the hospital course may allow old blood to continue to be expelled from the GI tract slowly throughout the hospital course, creating an ambiguous picture as to whether the patient has stopped bleeding or not. These ambiguous cases may result in inpatient colonoscopy procedures in patients who may otherwise be able to follow up safely in the outpatient setting. Frequently, cessation of bleeding in an otherwise stable patient may allow the provider to discharge a patient for an outpatient colonoscopy (or just clinical follow-up without performing colonoscopy at all if they have a prior diagnosis of diverticulosis), reducing the overall length of hospital stay and risk of hospital-associated complications. A study of 20,010 patients demonstrated that early colonoscopy for DB did not reduce the risk of recurrent bleeding after discharge and showed a slight risk of increased readmissions within 30 days [7]. Our results did not show increased readmission rates at 30 and 90 days post-discharge for EBP patients compared to LBP.

Additionally, our results suggest that EBP administration may result in reduced utilization of hospital resources such as unnecessary imaging studies, transfusions, and prolonged hospital stays. Although these data trended towards statistical significance, it did not reach it, presumably due to our small sample size. The incorporation of EBP administration in the management of patients with suspected acute DB may allow for earlier discharges and reducing resource consumption. One study from 2018 demonstrated that on average, the yearly cost of DB in the USA exceeds $500 million per year [8]. The increasing emphasis on proper utilization of healthcare resources continues to grow given our expanding and aging population, as the incidence of DB increases with age. The use of a technique such as EBP administration may help reduce additional imaging studies, such as nuclear medicine studies and CT angiograms, which carry their own risks of radiation exposure and nephrotoxicity in cases where bleeding has already stopped. EBP may actually be helpful in determining which patients have significant active ongoing bleeding after bowel prep and may benefit from additional imaging to identify a bleed source [9,10]. We suggest that EBP administration may be incorporated into standard practice by administering EBP within 24 hours to all patients presenting with suspected acute DB. The benefit of adopting this strategy would be that those patients in whom bleeding ceases after EBP will be identified earlier, whereas those patients in whom active bleeding continues will already be prepped for undergoing colonoscopic evaluation if needed in a timely manner.

Our study has several limitations: its retrospective nature, being conducted at a single tertiary care center with a limited sample size, inherently limits the generalizability of our results. The smaller sample size may have prevented some of the results from reaching statistical significance. Additionally, there is the limitation of potential bias from the physician determining the best choice for bowel preparation. Comparisons between variables such as length of stay, number of CT angiograms ordered, and timing to resolution of the bleed showed differences between groups favoring EBP administration; however, statistical significance was not achieved possibly for this reason. Further research is needed to confirm the results from this retrospective study. Furthermore, the retrospective nature of our study also introduces the possibility of selection bias, as the selection of patients for inclusion into each of the groups was done at the discretion of the attending physician. Additionally, there was a lack of control for specific comorbidities or anticoagulation status in the statistical analysis.

## Conclusions

The culprit lesion for GI bleeds can be difficult to determine, and DB is mostly a clinical diagnosis. Many providers find that it may be advantageous to forego colonoscopy and elect for observation and supportive care during the hospital stay among certain high-risk patient populations. This is especially true if the patient’s presentation is classic for DB and with documented prior diverticular disease concomitant with multiple comorbidities. The therapeutic utility of colonoscopy in addressing lower GI bleeds is low; therefore, reducing the number of unnecessary colonoscopy procedures in this demographic would be useful. Our study suggests that EBP provides the clinician with useful objective information about the rate of ongoing bleeding to significantly reduce colonoscopy performance in this setting. While each case requires evaluation of the entire clinical picture, our results demonstrate that EBP administration is a reasonable clinical strategy for those presenting with acute DB and may result in a significant decrease in health resource utilization. Further prospective studies are needed to examine and validate these findings.
